# Genotype-by-environment interaction of newly-developed sweet potato genotypes for storage root yield, yield-related traits and resistance to sweet potato virus disease

**DOI:** 10.1016/j.heliyon.2019.e01448

**Published:** 2019-03-29

**Authors:** Stephan Ngailo, Hussein Shimelis, Julia Sibiya, Kiddo Mtunda, Jacob Mashilo

**Affiliations:** a University of KwaZulu-Natal, School of Agricultural, Earth and Environmental Sciences, African Centre for Crop Improvement (ACCI), Private Bag X01, Scottsville, 3209, Pietermaritzburg, South Africa; b Sugarcane Research Institute, Kibaha, Tanzania

**Keywords:** Agriculture, Plant biology

## Abstract

Genotype-by-environment interaction analysis is key for selection and cultivar release, and to identify suitable production and test environments. The objective of this study was to determine the magnitude of genotype-by-environment interaction (GEI) for storage root yield, yield-related traits and sweet potato virus disease (SPVD) resistance among candidate sweet potato genotypes in Tanzania. Twenty-three newly bred clones and three check varieties were evaluated across six diverse environments using a randomized complete block design with three replications. The Additive Main Effect and Multiplicative Interaction (AMMI) and genotype and genotype-by-environment (GGE) biplot analyses were used to determine GEI of genotypes. Genotype, environment and GEI effects were highly significant (*P* ≤ 0.01) for the assessed traits. Further, AMMI analysis of variance revealed highly significant (*P* ≤ 0.001) differences among genotypes, environments and G × E interaction effects for all the studied traits. Both AMMI and GGE biplot analyses identified the following promising genotypes: G2 (Resisto × Ukerewe), G3 (Ukerewe × Ex-Msimbu-1), G4 (03-03 x SPKBH008), G12 (Ukerewe × SPKBH008) and G18 (Resisto × Simama) with high yields, high dry matter content and SPVD resistance across all test environments. The candidate genotypes are recommended for further stability tests and release in Tanzania or similar environments.

## 1 Introduction

Sweet potato (*Ipomoea batatas* [L.] Lam., 2n = 6x = 90) is an important storage root crop grown for diverse uses such as for food, feed and industrial raw material. It is a rich source of carbohydrates, vitamins A and C, fiber and minerals (Woolf, 1992; Teow et al., 2007). The crop has inherently low requirement of production inputs. Also it provides reasonable yields under marginal growing conditions making it the crop of choice widely cultivated in sub-Saharan Africa (SSA) (Karyeija et al., 1998). Sweet potato is cultivated on approximately 3.7 million hectares annually in SSA (FAOSTAT, 2013). However, the yield levels in the region are relatively low ranging between 4.0–10.0 tons/ha, compared with average yield of 21.5 tons/ha reported elsewhere (FAOSTAT, 2014).

Tanzania is the second largest producer of sweet potato in Africa. In the country, the average sweet potato yields are estimated at about 5 tons/ha (Ngailo et al., 2015). Low yields are attributed but not limited to unavailability of improved cultivars. Further the presently cultivated varieties are highly susceptible to viral diseases such as the Sweet Potato Virus Disease (SPVD) (Gibson et al., 1998; Gwandu et al., 2012). Gibson et al. (2000) reported that two viruses are prevalent in Tanzania namely: Sweet Potato Feathery Mottle Potyvirus (SPFMV) and Sweet Potato Chlorotic Stunt Virus (SPCSV). Hence dual-infection by SPFMV and SPCSV cause SPVD. In the country, farmers often acquire virus-free planting material of high-yielding sweet potato genotypes through the Quality Vines Project. However, farmers often re-plant infected vines harvested from previous crop or acquired from other farmers. These planting materials are mostly infected by SPVD causing devastating yield losses under severe epidemics (Rahma et al., 2015, 2018). This necessitates the need to develop high and stable yielding and SPVD resistant genotypes for sustainable production and productivity of the crop in Tanzania (Gibson et al., 2000; Ngailo et al., 2016; Kagimbo et al., 2018).

In an endeavor to develop high yielding and SPVD resistant genotypes, Ngailo (2013) developed 23 promising new clones after designed crosses involving complementary parents using a half diallel mating design (Hayman, 1954; Griffing, 1956). The newly developed clones were relatively high yielding (with storage root yields of >10 tons/ha) and resistant to SPVD (Ngailo, 2013). The candidate clones needed to be evaluated across target agro-ecologies in Tanzania to select high performing candidate varieties.

Genotype-by-environment interaction analysis is key for selection and cultivar recommendation, and to identify suitable production and test environments (Manrique and Hermann, 2000). Storage root yield and quality of sweet potato is prone to environmental changes resulting in variable yield and quality owing to genotype-by-environment interaction (Grüneberg et al., 2005; Nakitandwe et al., 2005; Laurie and Booyse, 2015; Gurmu et al., 2017). Genotype-by-environment interaction (GEI) leads to differencial responses of genotypes across growing environments and may limit selection response (Rukundo et al., 2013). Therefore, GEI analysis is an essential component in candidate variety evaluation that can lead to the release of high yielding and SPVD resistant sweet potato genotypes (Mwanga et al., 2009, 2011, 2016; Adebola et al., 2013; Shumbusha et al., 2014; Gurmu et al., 2017).

Statistical methods such as the Additive Main Effect and Multiplicative Interaction (AMMI) (Gauch, 1992) and genotype-by-environment interaction (GGE) biplot (Yan and Kang, 2003; Yan and Tinker, 2006) analyses are widely used in GEI analysis. The two methods have been previously employed in many sweet potato improvement programmes. For example, the AMMI model was successfully used for GEI and stability analysis among sweet potato clones across different environments in Turkey (Caliskan et al., 2007). Further, Laurie and Booyse (2015) used GGE biplots and identified suitable sweet potato genotypes and representative environments in South Africa. In light of the above background, the objective of this study was to determine the magnitude of GEI for storage root yield, yield-related traits and sweet potato virus disease resistance among candidate sweet potato genotypes.

## 2 Materials and methods

### 2.1 Study sites and planting materials

The study was conducted in six diverse and sweet potato growing environments in Tanzania namely: Gairo, Kilombero Agricultural Training Research Institute (KATRIN), Sokoine University of Agriculture (SUA), Sugarcane Research Institute (SRI), Chambezi and Mkuranga. The sites represent low to high altitudes with varied agro-ecological conditions. The study sites are among the major sweet growing areas and are hotspot areas for SPVD in Tanzania. The description of experimental sites and chemical composition of the soils at each site is presented in Table 1. Twenty-three experimental clones were selected from families developed through a diallel cross (Ngailo, 2013). The F1 progenies were originally field evaluated along with other three check varieties. The clones were selected based on various attributes including storage root flesh colour, dry matter content (DMC), fresh root yields and resistance to sweet potato virus disease (Table 2).

**Table 1:** Geographical and soil descriptions of the study environments.

Location	Location (environment) code	Coordinates		Altitude (masl)	Soil parameters								
					Textural class	pH (H2O)	OC (%)	TN (%)	Av. P (meq/100 g)	Exchangeable bases (meq/100 g)			
										Ca	Mg	K	N
Gairo	E1	E036o54′787″	S06o08′156″	1310	Sandy clay loam	5.9	0.81	0.08	5.9	4.1	1.9	0.66	0.20
SRI	E2	E038o58′315″	S06o46′701″	169	Clay	6.7	0.71	0.07	3.9	6.2	2.1	0.48	0.26
KATRIN	E3	E036o39′945″	S08o03′612″	288	Sandy loam	6.0	1.15	0.06	6.0	9.9	2.1	0.53	0.25
SUA	E4	E037o38′756″	S06o50′252″	518	Clay	5.3	2.10	0.11	5.3	5.1	2.5	0.95	0.30
Chambezi	E5	E038o28′59″	S06o33′302″	47	Loamy sand	6.4	0.39	0.05	6.4	2.7	0.7	0.24	0.21
Mkuranga	E6	E039o11′689″	S06o08′306″	119	Sandy loam	6.4	0.37	0.06	6.4	2.0	0.4	0.24	0.17

Masl = metres above sea level; meq 100 g^−1^ = milli-equivalent per 100 g of soil; Av. P = Available phosphorus; OC = organic carbon; TN = total nitrogen; Ca = calcium; Mg = Magnesium; K = potassium; Na = Sodium; KATRIN = Kilombero Agricultural Training and Research Institute; SRI = Sugarcane Research Institute; SUA = Sokoine University of Agriculture.

**Table 2:** Description of sweet potato genotypes used in the study.

Genotypes	Genotype code	Root flesh colour	Root DMC (%)	Storage root yield (t/ha)	Resistance to SPVD
Resisto × Ukerewe	G1	Yellow	35.7	14.6	Moderately resistant
Resisto × Ukerewe	G2	Orange	35.7	14.6	Moderately resistant
Ukerewe × Ex-Msimbu-1	G3	Cream	36.1	13.0	Resistant
03-03 × SPKBH008	G4	Cream	35.8	18.3	Resistant
Ukerewe × SPKBH008	G5	White	32.9	10.7	Resistant
Mataya x Gairo	G6	Yellow	36.1	12.3	Resistant
Simama × Ex-Msimbu-1	G7	Pale Orange	36.1	12.3	Resistant
SPKBH008 x Ex-Msimbu-1	G8	Cream	37.0	16.7	Resistant
Mataya × Ukerewe	G9	Cream	37.8	16.3	Resistant
Resisto × Simama	G10	Pale Orange	36.1	16.9	Resistant
Resisto × Simama	G11	Pale Orange	36.1	16.9	Resistant
03-03 × SPKBH008	D12	Orange	36.0	16.7	Resistant
Mataya × Gairo	G13	Orange	36.1	17.0	Resistant
Resisto × Gairo	G14	Orange	35.7	14.7	Resistant
Ukerewe × Simama	G15	Cream	39.6	15.9	Resistant
Mataya × Ukerewe	G16	Yellow	37.8	13.7	Resistant
Mataya × Resisto	G17	Orange	34.0	15.3	Resistant
Resisto × Simama	G18	Cream	35.6	21.7	Resistant
Ukerewe × Simama	G19	Cream	39.6	17.5	Resistant
03-03 × Ukerewe	G20	Yellow	38.2	14.9	Moderately resistant
03-03 × Resisto	G21	Orange	33.4	15.4	Resistant
Ukerewe × Gairo	G22	Cream	37.0	16.0	Resistant
SPKBH008 × Ex-Msimbu-1	G23	Cream	37.0	16.0	Resistant
Simama	G24	Cream	38.1	21.4	Resistant
Mataya	G25	Orange	33.1	15.5	Susceptible
Ukerewe	G26	–	40.7	10.5	Resistant

Sr. No = serial number; DMC = dry matter content; SPVD = sweet potato virus disease.

### 2.2 Experimental design and field establishment

The experimental genotypes and check varieties were evaluated using a randomized complete block design with three replications at each site. Experimental plots consisted of two rows of 6 m for each genotype. The intra-row and inter-row spacing were 0.3 m and 1 m, respectively. Four to six node cuttings were planted on ridges. Agronomic practices such as weeding and fertilization were followed per recommendation for sweet potato production in Tanzania.

### 2.3 Data collection

At harvest, storage roots were grouped into marketable and un-marketable, counted and their fresh weight (kg) per plot was recorded and later converted to tonnes per hectare (tons/ha). This provided storage root yield per hectare. The number of roots was expressed on a plant basis. From each plot, a sample of three to four storage roots were collected for dry matter content (DMC) determination. DMC was determined as described by Carey and Reynoso (1999) and Tairo et al. (2008) with some modifications. A sample of 200 g was chopped from undamaged roots for each entry in each replication. The samples were oven dried at 70 °C for 72 hours until constant weight. The dried samples were weighed using an electronic weighing scale to calculate dry matter content as a percentage of the fresh weight.

Reactions to SPVD were assessed visually at 60, 90 and 120 days after planting using a 1 to 5 scale, where 1 = no visible symptoms, 2 = mild symptoms (a few local lesions on a few leaves), 3 = moderate symptoms (mosaic symptoms on leaves), 4 = severe symptoms (mosaic symptoms with plants showing stunted growth) and 5 = very severe symptoms of purpling/yellowing of leaves, severe leaf distortion, reduced leaf size and severe stunting (Mwanga et al., 2013). The genotypes Mataya (G25) and Ukerewe (G26) were used as susceptible and resistant checks, respectively. The field trials were harvested 120 days after planting.

#### 2.3.1 Data analysis

##### 2.3.1.1 Analysis of variance

The data collected for number of storage roots, storage root yield, dry matter content and SPVD across six environments sites were subjected to combined analysis of variance (ANOVA) using Statistical Analysis System version 9.2 (SAS, 2008). Genotype by environment interaction effect were detected in ANOVA that led to the GEI analysis using Additive Main Effect and Multiplicative Interaction (AMMI) and genotype-by-environment interaction (GGE) biplot models.

##### 2.3.1.2 GEI analysis

The above data were analyzed using AMMI and GGE biplots using GenStat 17^th^ edition (Payne et al., 2014) to determine the effects of genotypes, environments and their interaction. The GEI analyses were conducted using AMMI (Kempton, 1984; Gauch, 1988; Gauch and Zobel, 1988; Yan et al., 2000, 2001; Yan, 2001).

The AMMI statistical model is given below;

$${\bar{Y}}_{ijk}=\mu +G_{i}+E_{j}+{\sum_{k=1}^{m}\lambda_{k}\alpha }_{ik}\gamma_{jk}+\rho_{ij}$$

where: ${\bar{Y}}_{ijk}$ = the yield of the i^th^ genotype in the j^th^ environment, G_i_ = the mean of the i^th^ genotype minus the grand mean, $E_{j}$ = the mean of the j^th^ environment minus the grand mean, $\lambda_{k}$ = the square root of the eigen value of the k^th^ IPCA axis, $\alpha_{ik}$ and $\gamma_{jk}$ = the principal component scores for IPCA axis k of the i^th^ genotypes and the j^th^ environment, $\rho_{ij}$ = the deviation from the model. According to Zobel et al. (1988), AMMI with only two interaction principal component axes could be the best predictive model. Hence, two IPCAs were adopted in this study in AMMI analysis. AMMI stability value (ASV) was calculated to quantify and rank genotypes. This was carried out using a formula suggested by Purchase (1997):

AMMI Stability Value (ASV) = $\sqrt{\left[{\left(\frac{SSIPCA1}{SSIPCA2}\left(IPCA1\right)\right)}^{2}+{\left[IPCA2\right]}^{2}\right]}$; where, $\frac{SSIPCA1}{SSIPCA2}$ represents the weighted value assigned to the first interaction principal component score due to its high contributions in the GE model, SSIPCA1 and SSIPCA2 are the sum of squares for IPCA1 and IPCA2, respectively, and IPCA1 and IPCA2 are the first and second IPCA scores for each genotype. The larger the ASV the more specifically adapted the genotype is to a certain environment and the smaller ASV indicates a more stable genotype across environments (Purchase, 1997; Farshadfar et al., 2011). The model for a GGE biplot (Yan, 2002; Yan et al., 2007) based on singular value decomposition (SVD) of *t* principal components is:

$${\bar{Y}}_{ij}-\mu_{i}-\beta_{j}=\sum_{k=1}^{t}\lambda_{k}\alpha_{ik}\gamma_{jk}+\varepsilon_{ij}$$

where: ${\bar{Y}}_{ij}$ is the performance of genotype *i* in environment *j*, μ is the grand mean, $\beta_{j}$ is the main effect of environment *j*, *k* is the number of principal components (PC); $\lambda_{k}$ is singular value of the *k*^th^ PC; and $\alpha_{ik}$ and $\gamma_{jk}$ are the scores of *i*^th^ genotype and *j*^th^ environment, respectively for PC_k_; $\varepsilon_{ij}$ is the residual associated with genotype *i* in environment *j*. AMMI and GGE biplot were performed using GenStat 17^th^ edition (Payne et al., 2014).

## 3 Results and discussion

### 3.1 Effects of environment, genotype and genotype × environment interaction

Analysis of variance for number of roots per plant, storage root yield, dry matter content and resistance to SPVD showed highly significant (*P* ≤ 0.001) differences among the six test environments and the tested genotypes (Table 3). Further, highly significant (*P* ≤ 0.001) genotype by environment interaction effect was observed for all studied traits and SPVD resistance implying differential genotypic performances across environments. Further, the AMMI analysis of variance in the current study revealed great contribution of environments (64%) and GE interactions (25%) for variation in storage root yield compared with the main effect of genotypes (11%). Gauch and Zobel (1997) reported that the main effect of environments represented about 80% of the total variation, whereas both genotype and G × E interactions effects represented 10% concurring with current findings. Gurmu (2015) also reported larger contribution of environmental (49.4%), genotypic (15.04%) and interaction (17.4%) for storage root yield in sweet potato in that order. Also, Kathabwalika et al. (2013) reported larger contribution of the interaction effect than genotypic and environment effects for variation in root storage yield agreeing with the present findings. The observed variation in storage root yield is useful for selection and recommendation of promising genotypes in sweet potato improvement programmes (Laurie and Booyse, 2015; Mwanga et al., 2016; Shumbusha et al., 2014). A larger contribution of genotypic effects compared to environment and interaction effects for dry matter content (DMC) was observed in the present study. Similar to the present study, Oduro (2013) and Gurmu (2015) reported larger contribution of genotypic effects compared to environment and interaction effects for dry matter content in sweet potato. The dominant contribution of genotypic effect suggests that environment had little effect on dry matter content among the studied sweet potato genotypes. Similarly, Chiona (2009) observed relatively small G × E interaction effects for DMC and suggested that selection for DMC improvement may be conducted only in selected representative environments. It has been reported that the influence of G × E interaction on nutritional traits such as DMC is smaller compared to root storage yield (Grüneberg et al., 2005) concurring with findings in the present study. The minimum impact of G × E on DMC observed in the present study suggested genetic gains for improving this trait is possible via selection supporting findings by Gurmu et al. (2017). Interaction principal component analysis axes (IPCA1 and IPCA2) sufficiently accounted for 71% of GE interaction for SPVD resistance in the current study (Table 4). The presence of G × E interaction is reportedly contributed to break down of resistance in improved varieties grown in agro-ecologies with high SPVD pressure (Gibson et al., 1998; Karyeija et al., 1998). In the present study, some tested sweet potato genotypes such as G3, G6, G14, G20 and G24 had high level of SPVD resistance (Table 2).

**Table 3:** Partial analysis of variance with mean square values and significance tests for number of roots, storage root yield, dry matter content and resistance to SPVD among sweet potato clones evaluated across six environments in eastern Tanzania.

Sources of variation	df	Mean squares			
		NRPP	Storage root yield	DMC	SPVD
Environment (E)	5	30.92***	2396.37***	430.26***	6.38***
Rep (Environment)	12	2.26*	28.69***	5.60^ns^	1.35***
Genotypes (G)	25	13.76***	83.79***	98.09***	2.78***
G × E	125	2.86***	38.06***	10.79***	0.78***
Error	300	1.18	10.43	6.13	0.4
Trial statistics					
Mean		3.61	10.69	35.97	1.62
CV (%)		30.08	30.21	5.65	39.22
R^2^ (%)		71.45	85.97	82.93	63.59
LSD (0.05)		1.34	3.99	2.51	0.78

df = degrees of freedom; *, *** = significant at 0.05 and 0.001, respectively; ns = non-significant at 0.05; CV = coefficient of variation; DMC = dry matter content; SPVD = sweet potato virus disease; LSD = least significant difference; NRPP = number of roots per plant; *R*^*2*^ = coefficient of determination.

**Table 4:** AMMI analysis of variance for number of roots per plant, storage root yield, dry matter content and SPVD among 26 sweet potato clones evaluated across six environments in eastern Tanzania.

Sources of variation	df	Mean squares			
		NRPP	Storage root yield	DMC	SPVD
Genotypes (G)	25	13.77***	83.9***	98.1***	2.78***
Environments (E)	5	30.38***	2396.2***	430.3***	5.39***
Block	12	2.34^ns^	28.7***	5.6^ns^	1.53***
Interactions (G × E)	125	2.71***	38.0***	10.8***	0.78***
IPCA1	29	5.57***	107.5***	19.5***	1.40***
IPCA2	27	2.52***	28.8**	13.0***	1.08***
Residuals	69	1.58	12.4	6.3**	0.41^ns^
Error	300	1.17	10.4	4.1	0.40

df = degrees of freedom; *, *** = significant at 0.05 and 0.001, respectively; ns = non-significant at 0.05; NRPP = number of roots per plant; DMC = dry matter content; SPVD = sweet potato virus disease; GE = genotype by environment; IPCA1 and IPCA2 = first and second interaction principal component analysis axes.

### 3.2 Mean performance and GGE biplot analysis of genotypes for yield and related traits and reaction to SPVD

#### 3.2.1 Number of roots per plant

Genotype G20 ranked the best across all environments with a mean number of roots per plant of 5.8 (data not shown). This genotype (G20) performed better than other genotypes in four environments namely E2 (SRI), E3 (KATRIN), E4 (SUA) and E6 (Mkuranga) with mean root number of 7.7, 7.1, 8.2 and 5.8, respectively. Further, genotypes such as G22, G3 and G2 also ranked best across all environments with mean roots per plant of 5.3, 4.5 and 4.6 in that order. These genotypes (G22, G3 and G2) also performed better in four environments namely E2 (SRI), E3 (KATRIN), E4 (SUA) and E6 (Mkuranga). The number of roots per plant in the selected clones is higher than some sweet potato landrace varieties and improved genotypes currently cultivated in Tanzania (Kagimbo et al., 2018). Marked differences in root number may be attributed to genotypic variations. Among environments, E2 was the best environment with the highest mean number of roots per plant of 4.5, whereas Gairo (E1) was the poor environment and recorded the lowest mean number of roots per plant of 2.7.

**Fig. 1:**
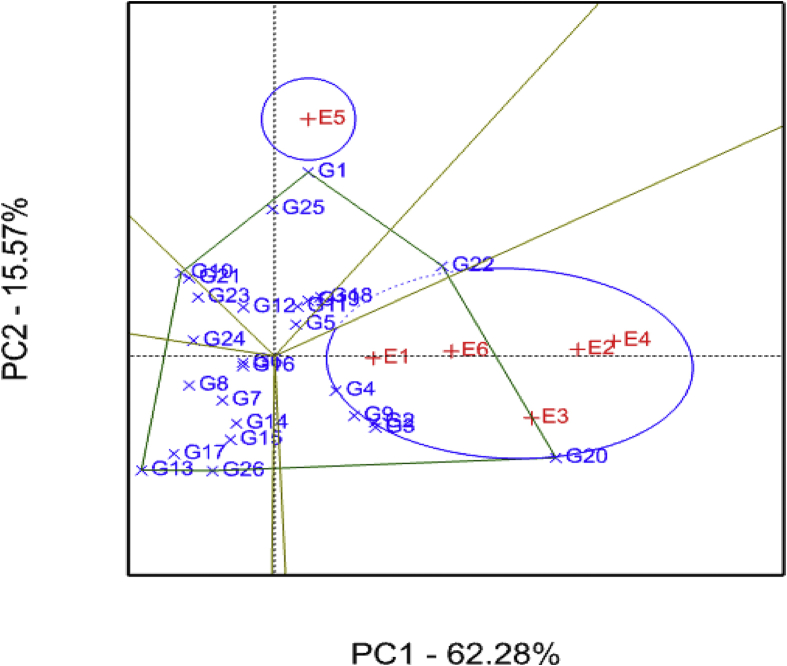
Number of roots per plant genotype plus genotype-by-environment (GGE) interaction biplot of PC1 vs PC2 showing the ‘which-won-where’ pattern of 26 sweet potato genotypes tested across six environments. See codes of environments in Table 1 and genotypes in Table 2.

GGE biplot analysis showing the relative performance of sweet potato genotypes for number of roots per plant across environments is presented in Fig. 1. The two principal component (PCs) explained about 78% of the total variation observed, of which PC1 and PC2 explained 62 and 16% of the total variation, respectively. Large and positive PC scores for a given genotype indicate a higher average value, while those with large negative PC scores imply a lower value (Yan et al., 2000). In the current study, genotypes G20, G22, G3, G2, G9 and G4 recorded the highest average number of roots per plant. Conversely, G13, G17, G10, G21 and G8 recorded the lowest mean number of roots per plant. Genotypes G6, G16, G5, G11 and G12 had low PC2 scores (i.e. close to zero) suggesting that they were more stable, useful for breeding. Similarly, genotypes at the vertices of the polygon (i.e. G1, G22, G20, G10 and G13) performed either best or poorest. Environments with large PC1 scores are better in discriminating the genotypes (Yan et al., 2000). Therefore, environments E2 (SRI) and E4 (SUA) discriminated the genotypes efficiently with regards to number of roots per plant. The which-won-where GGE biplots are divided by equality lines which aid in identification of mega-environments (Yan and Tinker, 2006) which identified two mega-environments in the current study.

**Fig. 2:**
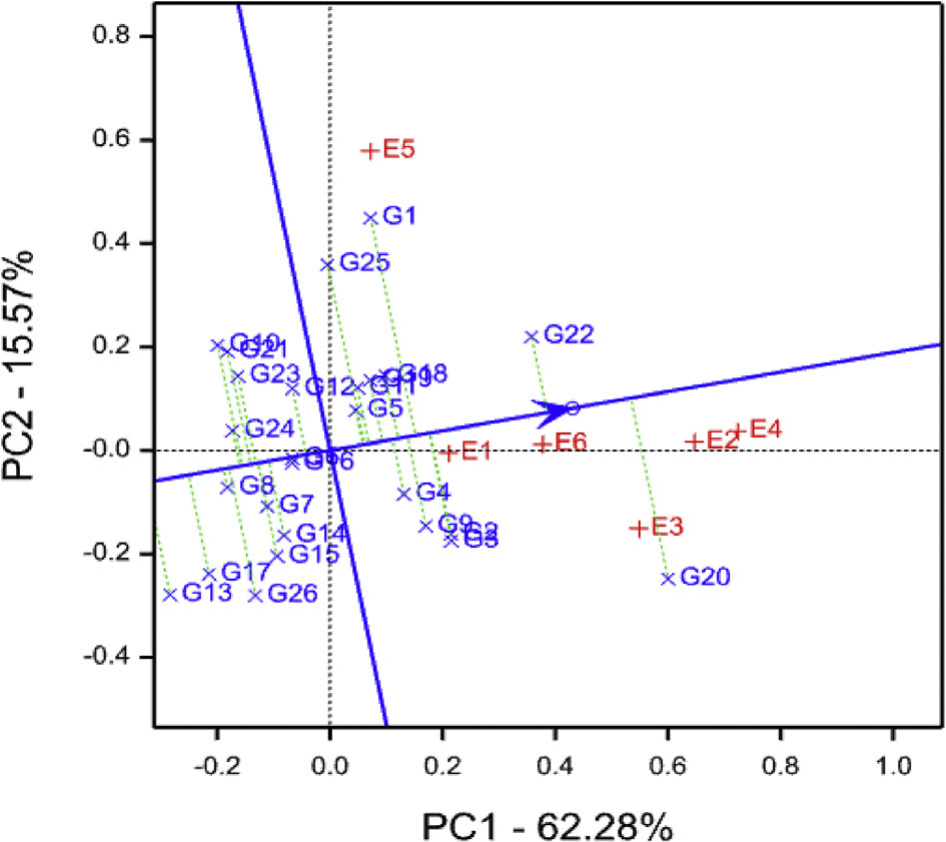
Number of roots per plant average-environment coordination view comparison biplot comparing 26 sweet potato genotypes tested across six environments See codes of environments in Table 1 and genotypes in Table 2.

The average-environment coordination (AEC) view of the GGE biplot comparing sweet potato genotypes across environments is shown in Fig. 2. Among the test environments, E5 (Chambezi) was highly variable compared to E1 (Gairo), E6 (Mkuranga), E4 (SUA) and E2 (SRI), which were relatively stable. Further, E6 was identified as the best representative environment for discriminating genotypes with respect to number of roots per plant, whereas E1 was the poorest. Furthermore*,* G22 was identified as the most desirable genotype for number of roots per plant due to its close proximity to the ideal variety (tip of arrow head on the vector through the average environment coordination in the center of inner circle).

#### 3.2.2 Storage root yield

Mean fresh root yield ranged from 7.5 to 17.2 tons/ha for G24 and G5, respectively with overall mean of 10.7 tons/ha (data not shown). More than 46% of the genotypes yielded above the overall mean with G5, G11 and G23 recording the highest storage root yields of 17.2, 13.8 and 13.5 tons/ha, respectively. Storage root yield of the identified clones is higher than those recently developed in Rwanda (Rukundo et al., 2017) and sweet potato genotypes released in sub-Saharan Africa (Mwanga et al., 2009; Tumwegamire et al., 2011). However, yield levels are lower than those reported by Kagimbo et al. (2019) in Tanzania. The yield levels of the presently selected sweet potato clones is higher than the mean yield of 5.3 tons/ha presently reported in the country (FAOSTAT, 2018). These clones serve as useful genetic resource for sustainable sweet potato production and for breeding. Genotypes G7, G12, G16, G22, G24, G25 and G26 recorded root yield <10 tons/ha which was below average. These suggest future breeding using these clones may result in limited yield improvement. Among the test environments, Mkuranga (E6) and KATRIN (E3) had the lowest and highest mean yields of 5.6 and 21.6 tons/ha, respectively. The genotypes G24 and G5 had the highest and lowest IPCA1 scores for root yield, respectively.

**Fig. 3:**
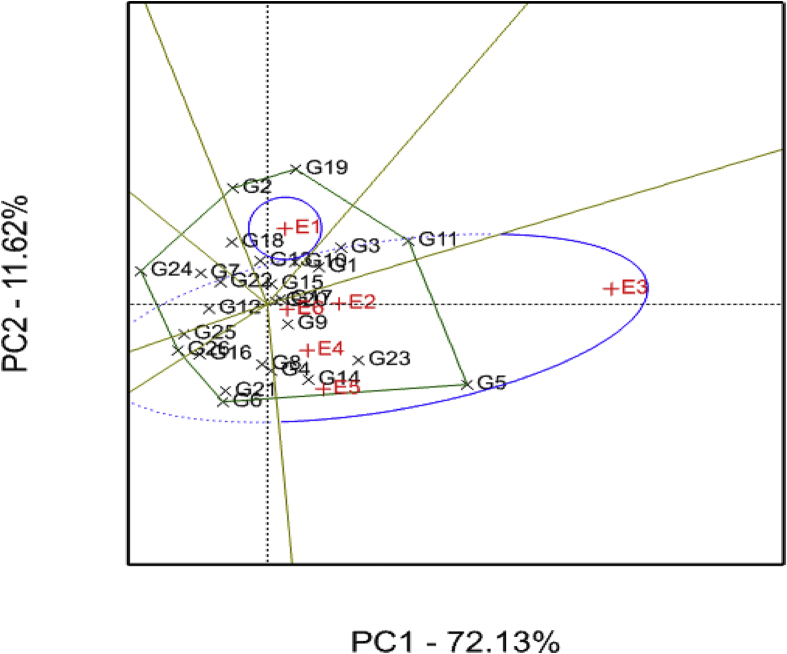
Storage root yield genotype plus genotype-by-environment (GGE) interaction biplot of PC1 vs PC2 showing the ‘which-won-where’ pattern of 26 sweet potato genotypes tested across six environments. See codes of environments in Table 1 and genotypes in Table 2.

The GGE biplot showing the “which–won- where” for storage root yield is presented in Fig. 3. The two PCs explained about 84% of the total variation observed. PC1 accounted for 72% of the total variation, whereas PC2 explained 12% of the total variation (Fig. 3). The GGE biplot revealed the genotypes G5, G11 and G19 as high yielding and responsive genotypes located at the vertices of the polygon agreeing with previous reports (Yan et al., 2000; Yan and Kang, 2003; Yan and Tinker, 2006). The identified genotypes are recommended for their specific adaptation (Fig. 3). For example, G5 performed well across most of the test environments, whereas G19 performed better at E1 (Gairo). Environment-specific adapted (i.e. unstable and responsive) varieties have the advantage to respond to environmental changes compared to widely-adapted (stable and non-responsive) varieties (Laurie and Booyse, 2015). Further, such genotypes have considerable yield advantage over widely-adapted stable genotypes in low-yielding environments (Grüneberg et al., 2005). On the contrary, genotypes G5, G7, G9, G17, G10 and G22 were the most stable across the test environments (Fig. 3) useful for breeding for yield stability in sweet potato improvement programmes. Additionally, two categories of genotypes with regards to storage root yield were identified in the present study namely: (i) high yielding genotypes with broad adaptation such as G5, G11 and G19 and (ii) high yielding genotypes with narrow adaptation such as G3, G10, G9, G14 and G20 (Fig. 3).

The GGE biplot identified two mega-environments with E1 (Gairo) constituting one mega-environment and E2 (SRI), E3 (KATRIN), E4 (SUA), E5 (Chambezi) and E6 (Mkuranga) composing the second mega-environment (Fig. 3), implying that only two sites, one from each mega-environment were discriminative of the tested genotypes for root storage root yield in agreement with Yan and Rajcan (2002) and Yan and Tinker (2006). Further, the identified mega-environments displayed different high yielding genotypes thus indicating presence of cross-over G × E interaction and inconsistent performance of the test genotypes across environments concurring with Gauch and Zobel (1997). KATRIN (E3) provided the highest storage root yield of 22 tons/ha and considered to be a good environment for testing of sweet potato genotypes for storage root yield.

**Fig. 4:**
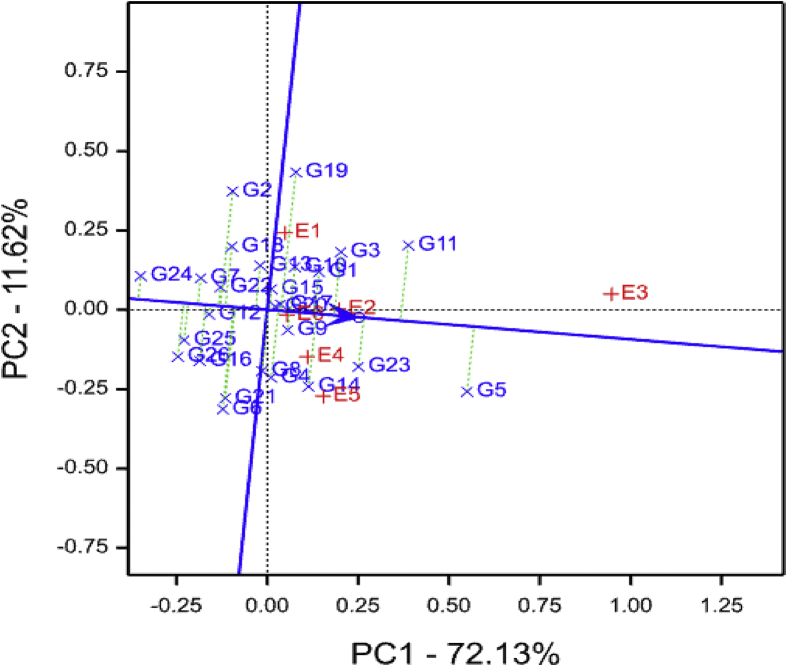
Storage root yield average-environment coordination view comparison biplot comparing 26 sweet potato genotypes tested across six environments. See codes of environments in Table 1 and genotypes in Table 2.

The AEC comparison using GGE biplot in the current study identified E6 (Mkuranga) and E2 (SRI) as the best representative or “ideal” environment for discriminating the tested genotypes with respect to storage root yield (Fig. 4). An “ideal” environment should be both discriminating of the genotypes and representative of the mega-environment (Yan, 2002). Further, the closer an environment is to the “ideal environment”, the better it is as a test environment (Yan, 2001). Similarly, the present study identified genotypes G9, G15, G17 and G20 as the most desirable genotypes for storage root yield (Fig. 4). Based on the AEC view comparison GGE biplot, an ideal genotype is associated with the greatest vector length of the high-yielding genotypes, and a desirable genotype is the one that is located closer to an ideal genotype (Yan, 2001). Four genotypes (G9, G15, G17 and G20) recorded high storage root yields of 12.1, 10.7, 10.8 and 11.0 tons/ha in that order across the test environments and may be recommended for cultivation to increase yield levels in all agro-ecologies of Tanzania. Root flesh colour among these genotypes (G9, G15, G17 and G20) varied from cream-fleshed and orange-fleshed types and exhibited high level of resistance to SPVD (Table 2). The release of these four cultivars may provide consumers with high levels of provitamin A contents to alleviate the widespread vitamin A deficiency present in Tanzania and other SSA countries.

#### 3.2.3 Dry matter content

Dry matter content (DMC) varied from 30.3 to 40.8% for genotypes G25 and G26 (check varieties), respectively, with an average of 36%. Among the newly bred genotypes, G1, G2, G5, G14 and G22 recorded the lowest DMC of 33.7, 32.9, 33.0, 32.6 and 33.6%, respectively which were below the average DMC. Conversely, G10, G13, G15 and G19 recorded high DMC of 38.1, 37.6, 37.6 and 38.7%, respectively. These are recommended for breeding for enhanced dry matter content. Breeding for high dry matter content is an important goal in sweet potato improvement programmes. In the present study, DMC of sweet potato clones was higher than those reported for sweet potato genotypes released previously in sub-Saharan Africa (Mwanga et al., 2009; Tumwegamire et al., 2011; Musembi et al., 2015). Test environments E1 (Gairo) and E5 (Chambezi) had the lowest and highest DMC of 32% and 38.2%, respectively.

**Fig. 5:**
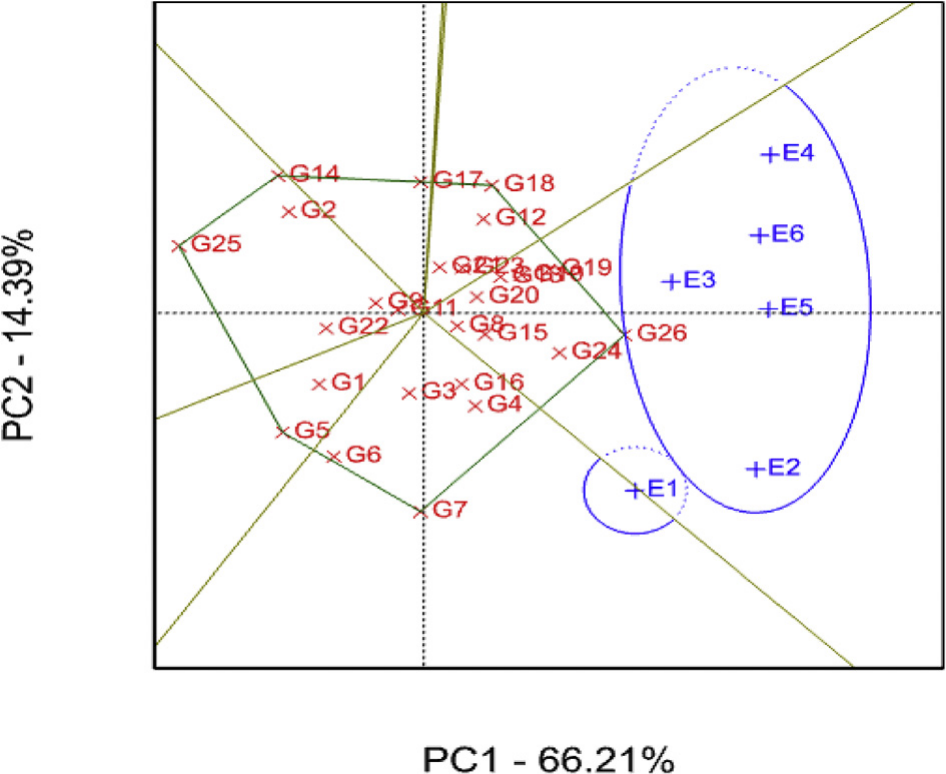
Dry matter content genotype plus genotype-by-environment (GGE) interaction biplot of PC1 vs PC2 showing the ‘which-won-where’ pattern of 26 sweet potato genotypes tested across six environments. See codes of environments in Table 1 and genotypes in Table 2.

GGE biplots showing “which-won-where” or which genotypes are best for which environment for dry matter content (DMC) is presented in Fig. 5. Both PC1 and PC2 accounted for about 81% of the total variation, implying that they sufficiently explained the GGE. PC1 accounted for 62% of the total variation, whereas PC2 explained 14% of total variation. Breeding for high dry matter content is an important breeding priority in sweet potato improvement programmes (Shumbusha et al., 2014). The GGE biplot in the current study revealed genotypes such as G26, G24, G19, G10 and G13 as relatively stable for dry matter content with relatively low PC2 scores. Further, G24 and G26 were considered the most stable genotypes with high DMC. DMC is less influenced by the environment (Grüneberg et al., 2005) suggesting that the identified clones are useful candidates for future breeding of sweet potato with enhanced DMC. Genotypes G7, G26, G18, G14, G25 and G5 were identified as highly responsive and prone to environment changes (Fig. 5) and therefore identified as “winning” genotypes in specific environments. Apart from possessing high dry matter contents (≥30%), the identified genotypes produced acceptable yield levels (≥8 tons/ha).

**Fig. 6:**
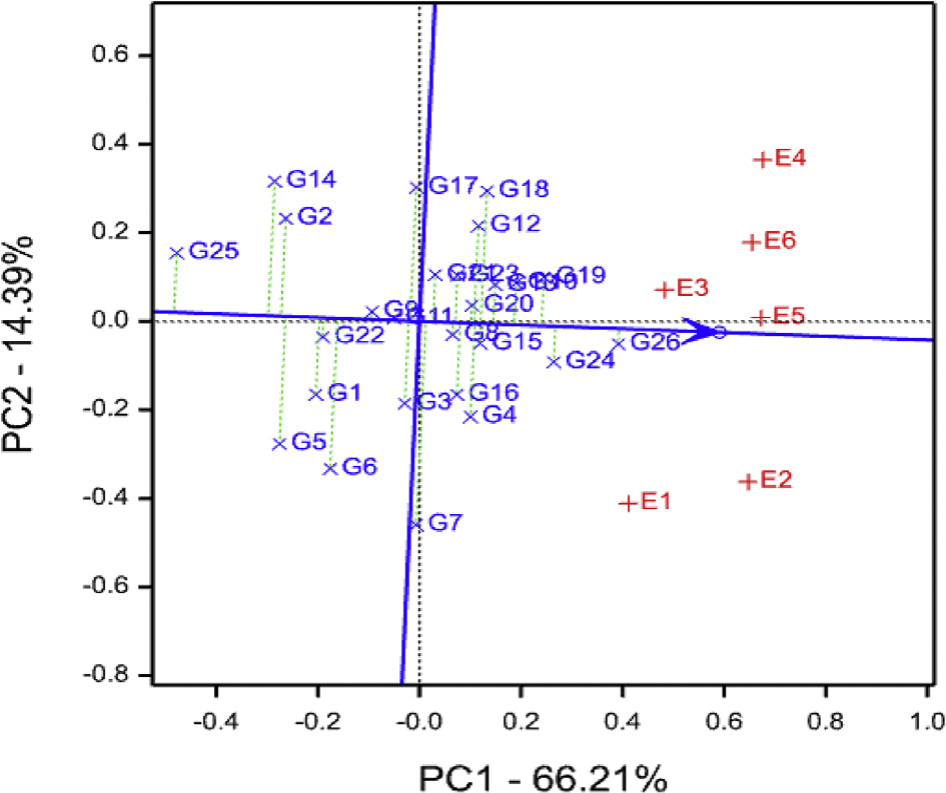
Dry matter content average-environment coordination view comparison biplot comparing 26 sweet potato genotypes tested across six environments. See codes of environments in Table 1 and genotypes in Table 2.

The AEC comparison view GGE biplot identified KATRIN (E3) and Chambezi (E5) as “ideal” environments for discriminating genotypes with respect to DMC, whereas environments such as E1 (Gairo) and E4 (SUA) as “non-ideal” environments (Fig. 6). Similarly, G26 was identified as the most desirable genotype with dry matter content of 40.8% and ASV value of 0.48 suggesting it was also relatively stable. Similarly, newly-developed genotypes such as G24 and G19 were also identified as ideal genotypes with dry matter contents of 38.9 and 38.7%, respectively. According to Yan and Tinker (2006), genotypes exhibiting both high mean performance and high stability across environments are qualified as ideal genotypes.

#### 3.2.4 Sweet potato virus disease resistance

The SPVD scores for the six environments varied from 1.17 to 3.11 corresponding for genotypes G4 and G25, respectively. About 62% of the genotypes recorded SPVD scores less than the average across all the six environments. Genotypes such as G4, G5, G7, G8, G9, G11, G12, G13, G17, G18, G21, G23 and G26 exhibited SPVD values of ≤1.5, suggesting their resistance to SPVD. The level of SPVD resistance among the presently tested sweet potato clones is comparable and in some instances higher than previously released commercial cultivars and clones (Gwandu et al., 2012; Shumbusha et al., 2014; Mwanga et al., 2016; Gurmu et al., 2018).

**Fig. 7:**
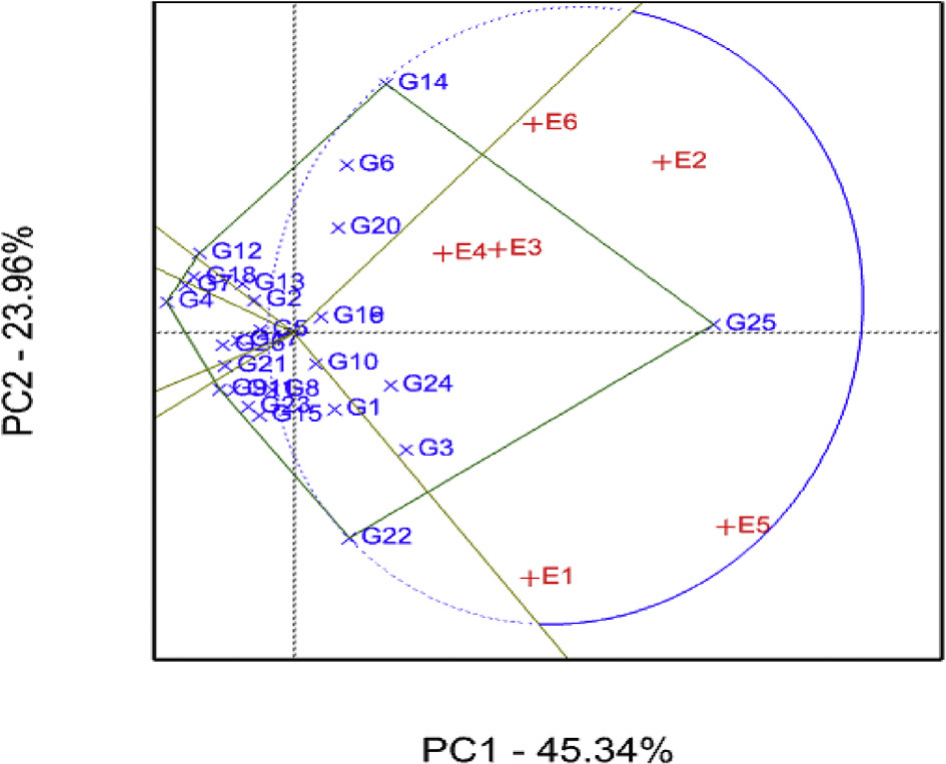
Sweet potato virus disease genotype plus genotype-by-environment (GGE) interaction biplot of PC1 vs PC2 showing the ‘which-won-where’ pattern of 26 sweet potato genotypes tested across six environments. See codes of environments in Table 1 and genotypes in Table 2.

The GGE biplot showing environments and respective sweetpotato genotypes for resistance to sweet potato virus disease is presented in Fig. 7. The two PCs accounted for 69% of the total variation with PC1 and PC2 accounting for 45% and 24% of total variation, respectively. With regards to SPVD resistance, genotypes with high and positive PC1 scores imply that they were most susceptible and those with negative PC1 are most resistant. SPVD is a major constraint to sweet potato production in sub-Saharan Africa (Gibson et al., 1998). SPVD prevalence is further exacerbated by the cultivation of susceptible varieties and a lack of effective control measures which further contributes to low sweet potato yields in the region. Development of SPVD resistance sweet potato genotypes with high yield potential are required to increase sweet potato production in SSA (Ngailo et al., 2016; Rukundo et al., 2017). The GGE biplot identified genotypes G2, G3, G4, G5, G7, G12 and G18 as resistant to SPVD with the lowest PC1 scores (Fig. 7). Further, seven genotypes (G2, G3, G4, G5, G7, G12 and G18) with SPVD resistance recorded root yield levels of ≥ 8 tons/ha and dry matter contents of ≥32%. Results of the present study suggested breeding sweet potato genotypes with high levels of resistance to SPVD combined with high yield and dry matter content is possible. Cultivation of the identified genotypes may be recommended to increase sweet potato production in Tanzania. Findings of this study agree with previous reports which developed sweet potato genotypes combining SPVD resistance with high yield potential and dry matter content (Mwanga et al., 2009, 2011, 2016; Shumbusha et al., 2014). The genotypes indicated as G10, G16, G19, G6, G26, G21, G11, G17 and G8 were stable for SPVD resistance across test environments, useful for breeding for SPVD stability. Amongst environments, E2 (SRI) and E5 (Chambezi) had large PC1 scores and can be considered good environments for testing of sweet potato genotypes for SPVD reaction (Fig. 7). Four genotypes: G4, G7, G12 and G18 were identified as the stable performers with SPVD resistance (Fig. 7). Among test environments, E3 (KATRIN) and E4 (SUA) were relatively the most representative environments in discriminating genotypes for SPVD resistance. The test environments had positive PC1 scores and constituted one mega-environment, hence they were similar in discriminating genotypes for SPVD resistance. However, E3 (KATRIN) and E4 (SUA) were relatively the most representative environments in discriminating genotypes for SPVD resistance.

**Fig. 8:**
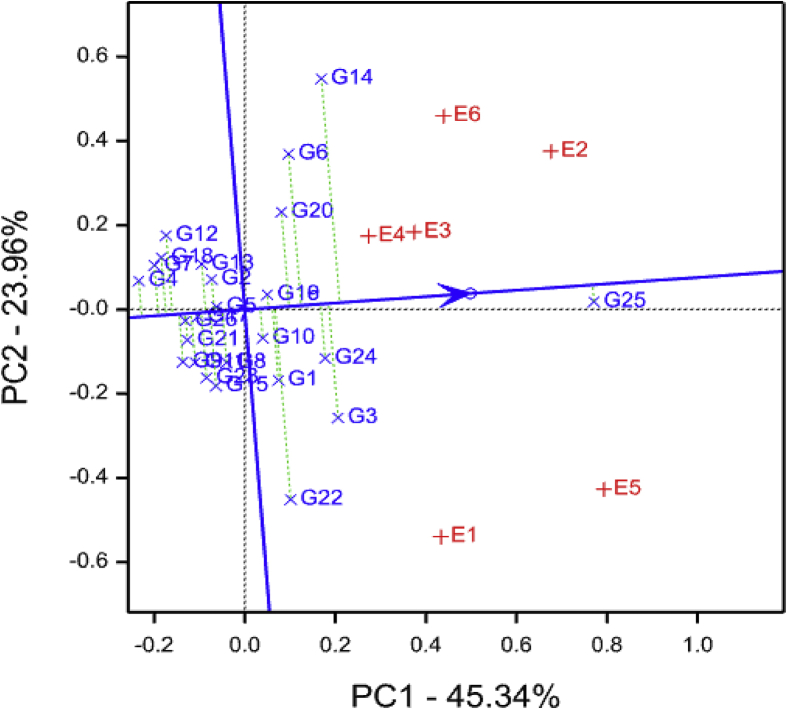
Sweet potato virus disease average-environment coordination view comparison biplot comparing 26 sweet potato genotypes tested across six environments. See codes of environments in Table 1 and genotypes in Table 2.

The AEC view of the GGE biplot further indicated that KATRIN (E3) and SUA (E4) were identified as the best representative environments for discriminating genotypes with respect to SPVD, whereas E1 (Gairo) and E6 (Mkuranga) and E1 (Gairo) were the poorest (Fig. 8). Genotypes G4 and G7 were identified as the most desirable genotypes for SPVD resistance and G25 the least desirable.

## 4 Conclusion

The current study determined the magnitude of genotype-by-environment interaction and stability for number of roots per plant, storage root yield, dry matter content and reaction to sweet potato virus disease among newly developed sweet potato genotypes in eastern Tanzania. Candidate sweet potato genotypes G2 (Resisto × Ukerewe), G3 (Ukerewe × Ex-Msimbu-1), G4 (03-03 x SPKBH008), G12 (Ukerewe × SPKBH008) and G18 (Resisto × Simama) are good yielders with high dry matter content and SPVD resistance across all test environments. The candidate genotypes are recommended for further stability tests and release in Tanzania or similar environments.

## Declarations

### Author contribution statement

Stephan Ngailo: Conceived and designed the experiments; Performed the experiments; Analyzed and interpreted the data; Contributed reagents, materials, analysis tools or data; Wrote the paper.

Hussein Shimelis, Julia Sibiya, Kiddo Mtunda: Conceived and designed the experiments; Analyzed and interpreted the data; Wrote the paper.

Jacob Mashilo: Analyzed and interpreted the data; Wrote the paper.

### Funding statement

This work was supported by the Alliance for a Green Revolution in Africa (AGRA) through financial support of the study through the African Centre for Crop Improvement (ACCI).

### Competing interest statement

The authors declare no conflict of interest.

### Additional information

No additional information is available for this paper.
